# Novel near-point of care nucleic acid amplification tests (NPOC NAAT) for TB: addressing readiness at primary health care

**DOI:** 10.5588/ijtldopen.26.0424

**Published:** 2026-11-13

**Authors:** L.E. Scott, G. Theron, A. David, R. Venter, N. Martinson, on behalf of the NPOC NAAT TB Implementation Readiness Group

**Affiliations:** 1 WITS Diagnostic Innovation Hub, Health Science Research Office, Faculty of Health Science, University of the Witwatersrand, Johannesburg, South Africa;; 2 DSI-NRF Centre of Excellence for Biomedical Tuberculosis Research, South African Medical Research Council Centre for Tuberculosis Research, Division of Molecular Biology and Human Genetics, and Biomedical Research Institute, Faculty of Medicine and Health Sciences, Stellenbosch University, Cape Town South Africa;; 3 Perinatal HIV Research Unit (PHRU), Faculty of Health Sciences, University of the Witwatersrand, Johannesburg, South Africa.

**Keywords:** tuberculosis, Pluslife MiniDock MTB, implementation, biosafety

Dear Editor,

Near-point of care (NPOC) nucleic acid amplification tests (NAATs) for TB are finally a reality – recently approved by the WHO and being investigated for use in high-burden countries, including South Africa. They offer the promise of cheap and accurate diagnosis at the facility where the patient presents, with rapid result turnaround enabling same-day treatment initiation. Crucially, tongue swab-based testing extends this capability to people unable to produce sputum. The greatest potential impact lies in reducing pre-treatment loss-to-follow-up^1^ largely due to the need to return for a second visit. The availability of NPOC tests may also increase the propensity to test. The MiniDock MTB (Guangzhou Pluslife Biotech Co., Ltd) test is the first WHO-recommended NPOC NAAT for rapid TB diagnosis,^2^ and it is uniquely designed for direct testing of both tongue swabs and sputum-dipped swabs. Reported diagnostic accuracy is promising with tongue swab sensitivity of 75%–86% and sputum swab sensitivity from 85% to 91% and specificity of 96%–100%, the latter being comparable to sputum-based Xpert MTB/RIF Ultra which showed a sensitivity of 89%–100% and specificity of 97%–99%.^2–5^ Some studies do not specify whether sputum swabbing was performed at point-of-care (POC) or in laboratory settings, a distinction with operational relevance due to potential for aerosol generation and droplet dispersal of viable *Mycobacterium tuberculosis* complex prior to inactivation or thermolysis (as illustrated in the Figure).

**Figure.**
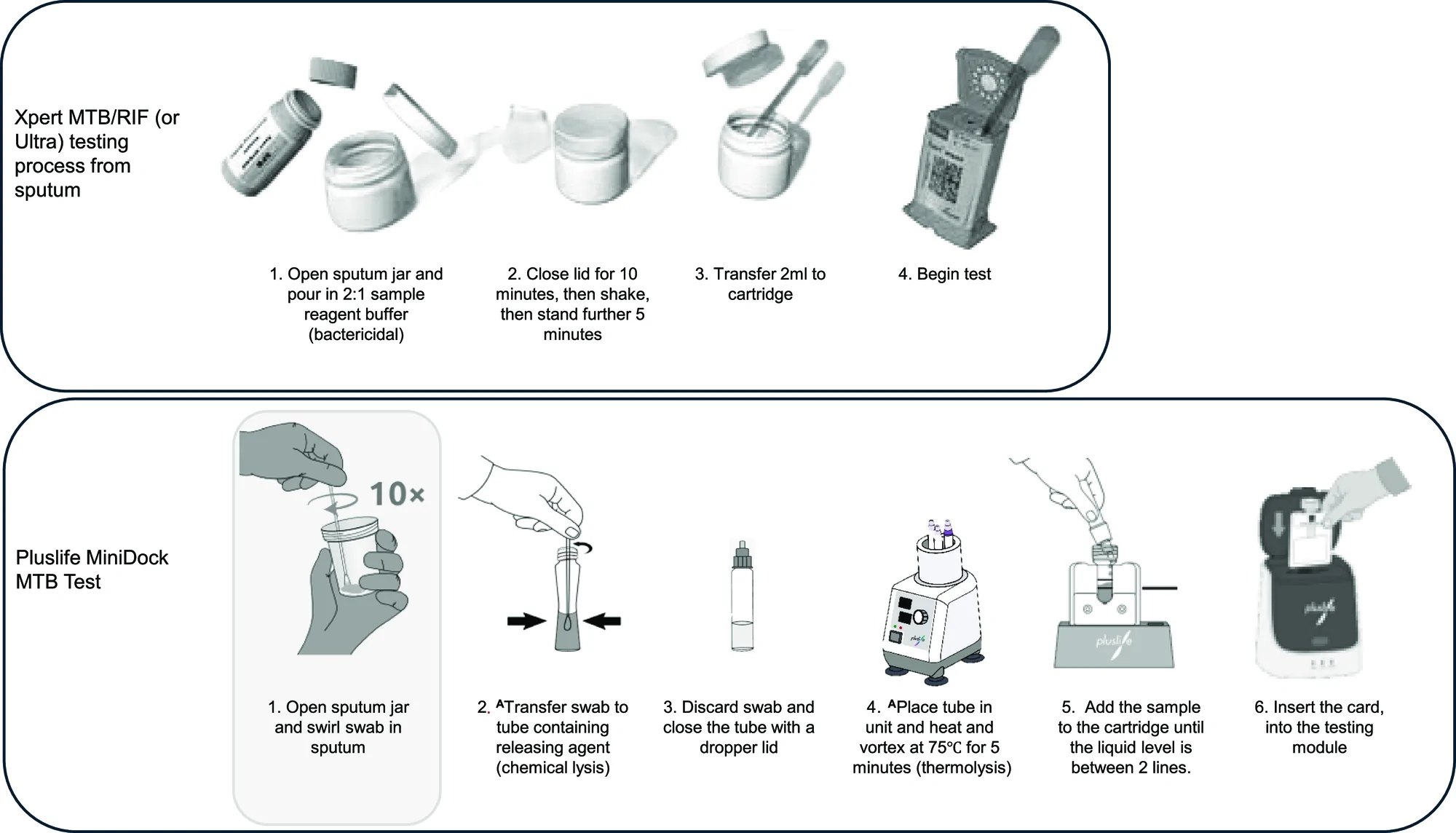
Schematic of sputum testing process for the Xpert MTB/RIF (or Ultra) and the Pluslife MiniDock MTB Test. The grey box in step 1 of MiniDock MTB sputum processing highlights the risk of *Mycobacterium tuberculosis* complex aerosolisation. Images were modified from supplier training materials. ^**A**^It is critical that both processing steps (steps 2 and 4) are performed.

According to the WHO, POC testing carries a low biosafety risk similar to smear microscopy.^6^ However, the quantity and infectiousness of aerosols generated during open-container sputum swabbing is unknown and needs to be assessed. Despite these promising diagnostic accuracy data, and recent evidence of non-inferiority for diagnostic yield,^7^ tongue swab performance in paucibacillary disease remains a critical evidence gap. In a multicentre study, tongue swab sensitivity ranged from 98% in high-load specimens to 48% in very-low-load specimens, compared with 100% and 87%, respectively, on sputum swab testing.^3^ Performance in paucibacillary populations such as those in community-based screening, people with HIV (PHIV), and children remains insufficiently characterised and warrants dedicated evaluation.^8^ With WHO recommendation granted and early adopter countries moving toward programmatic adoption, operational guidance for safe implementation in non-laboratory settings is urgently needed.

Between April and May 2026, we constituted an international expert group – the NPOC NAAT TB Implementation Readiness Group – comprising specialists in NAAT evaluation, implementation science, clinical usability, and aerobiology. One of the concerns driving this work is the biosafety risk specific to the MiniDock sputum swab workflow: unlike existing TB NAAT platforms, where sputum is chemically inactivated before manipulation, the MiniDock workflow requires swabbing raw sputum in an open container. Although reported before the availability of NPOC sputum swab testing, Banada et al. demonstrated that the preparation of acid-fast smears from sputum samples spiked with *Mycobacterium bovis* BCG at 5 × 10^8^ CFU/ml produced 16 and 325 CFU/m^3^ air as measured by an Andersen impactor and a BioSampler, respectively. By comparison, the sample preparation steps and processing for the Xpert assay did not produce any culturable bioaerosols.^9^ Guided by the Stop TB Partnership and Global Laboratory Initiative NPOC toolkit^10^ and considerations raised by Denkinger et al.,^11^ the group identified the following operational issues to be addressed:

1. Identifying the appropriate cadre of staff to expand access to NPOC testing: addressing scope of practice, role clarification, and training needs, and specifically, the extent to which non-laboratory staff can undertake this role (including troubleshooting).
2. Biosafety and infection control: managing sputum sampling by non-laboratory-trained staff, assessing risks of aerosolisation during test procedures, risks and management of spillage, potential for cross-contamination, specimen mix-up, PPE supply, infrastructure ventilation needs (including exhaust ventilation and ultraviolet lighting), and waste disposal.
3. Workflow: defining where sputum and tongue sampling takes place and whether patients can perform sputum swabbing on expectorated specimens, and the potential impact of sputum quality and volume on the latter – while maintaining proximity between collection and testing to minimise patient disengagement and address stigma.
4. Result management: linking patient samples to test results, and reporting results to enable same-day linkage to care and for monitoring and evaluation purposes.
5. Quality assurance – monitoring and maintaining quality in multiple decentralised sites, proficiency assessment, and post-market surveillance.
6. Ensuring confirmatory and/or drug susceptibility testing on the residual sputum (adequate volume, disposition) and the impact of biomass depletion from swabbing.
7. Appropriate waste management, which appears manageable within current practices.

These issues, together with an assessment of the aerosolisation risk, should be addressed as a priority in demonstration studies. Two areas of innovation may help address biosafety concerns: 1) Engineering controls to reduce exposure risk during sputum swabbing, including mechanical barriers such as innovative sputum cup or lid re-designs, containment hoods, and plastic enclosure systems. 2) Pre-swab sputum decontamination: inactivation of sputum prior to swabbing requires evaluation, with the caveat that reagents must not compromise MiniDock MTB assay performance or produce dilution effects that impair test performance. In addition to these innovations, existing and practical biosafety measures that can be implemented in peripheral health care settings where NPOC testing is expected to occur must also be considered. These may include appropriate specimen handling procedures, optimisation of ventilation, use of appropriate PPE, staff training and competency, and other practical risk-mitigation strategies designed to minimise exposure to potentially infectious aerosols.

The group emphasised modifying implementation guidance to ensure adaptability to different settings (such as including end-user-focused training materials with quick reference guides and visual aids), while recognising that adequate training and change management are of paramount importance for successful implementation. Diagnostic network optimisation will be required to fully integrate the technology into multi-platform diagnostic networks. Many of the operational challenges identified are not unique to NPOC NAAT TB testing and will require broader consideration by developers, and implementers, seeking to leverage the multi-disease capabilities of emerging NPOC NAAT platforms, including applications for sexually transmitted infection and HPV testing. Evaluation of MiniDock MTB performance in paucibacillary populations (PHIV, children, and individuals identified through community-based screening) is required as evidence remains insufficient to guide programmatic use. Addressing these gaps will require coordinated action from manufacturers, WHO, funders, and national programmes to translate the promise of NPOC NAAT testing into equitable, safe, and effective implementation at scale.
